# An Empirical Analysis of Delayed Monthly Bill Payments as an Early Risk Factor of Increased Suicidal Behavior

**DOI:** 10.3390/ijerph16162929

**Published:** 2019-08-15

**Authors:** Sujin Kim, Myoungsoon You

**Affiliations:** 1 Department of Health Care Policy Research, Korea Institute for Health and Social Affairs, Sejong 30147, Korea; 2 Department of Public Health Science, Graduate School of Public Health, and Institute of Health and Environment, Seoul National University, 1 Gwanak-ro, Gwanak-gu, Seoul 08826, Korea

**Keywords:** suicidal behavior, suicidal ideation, suicide attempts, late payments, financial hardship, South Korea

## Abstract

This study examines the potential of delayed monthly bill payments as a predictor of suicidal behavior in South Korea with the highest suicide rate among developed countries. Using the Korea Welfare Panel Study, multivariable logistic regressions examined the association between suicide ideation/attempts and the frequency of late payments on utility bills or National Health Insurance premium during last three years. Confounding factors such as past depression and suicide ideation/attempts history were adjusted for. Among 10,988 individuals, 2.7% reported suicide ideation and 0.11% attempted suicide in the past year, while 7.1% reported they paid late once or more during the last three years. Adults with two or more delayed payment had 2.32 times increased odds of suicidal ideation and 10.99 times increased odds of suicide attempts, compared to having no late payments. Adjusting for other socio-economic characteristics rarely changed the relationship between late payments and suicide ideation/attempts (for suicidal ideation, OR = 2.11; for suicide attempts: OR = 7.44), suggesting the independent effect of late payments on suicide behavior. With these findings, it can be suggested that late payment is an important factor, preemptively signaling suicide behavior with serious consequences in health and life.

## 1. Introduction

Suicide is one of the leading causes of death. More than 800,000 people around the world die by suicide every year, and the trend is far from decreasing [1]. There are countries such as South Korea where suicide is a particularly serious public health problem. For a decade, South Korea has remained the highest suicide rate among the member countries of Organization for Economic Co-operation and Development (OECD): The rate was 33.8 per 100,000 in 2009 and has decreased slightly to 24.6 in 2017 while OECD average was 12.0 [2].

In the literature, suicidal behavior, including both suicidal ideation and attempt, has been associated with psychiatric and socioeconomic factors [3,4]. On the one hand, the presence of mental disorder is identified as the key risk factor of suicide [5]. A meta-analysis study, however, found the effects were weaker than expected, showing ORs of less than 2.0 for suicide behavior and death [6]. On the other hand, the findings of the negative association between suicide and economic condition have been accumulated [7,8,9,10,11,12,13,14]. These studies highlight that medical approaches have limitations in further investigating issues in relation to underlying motivations such as unemployment and low income. However, the remarkably high suicides in South Korea call for more preemptive efforts in research and policy. This means the necessity of evidence of a risk factor not only with a strong association with suicidal behavior, but also a potential for early detection and intervention of suicide behavior [15]. 

As a response, this study aims to investigate the relation between deteriorating economic condition in an individual’s daily life and suicidal behavior among the Korean population. A special focus is upon late payment of monthly bills (i.e., utility and health insurance). It is assumed that repeated delay of bill payment may impact on individuals’ psychological status and be associated with the development of an adjustment disorder [16,17,18]. If the payment delay continues, the repeated experience can increase stress that there would be no way to be bailed out. In addition, the repeated failure in small bill payments is likely to increase feelings of unhappiness and stimulate extreme thinking of avoidance or exit. 

Despite the importance of this topic, few studies address the issue of late payments and suicide [19]. Further, if the importance of late payments in predicting suicide behavior is statistically supported, it could save ‘golden time’ for preventing suicidal behavior. The variables such as unemployment and poverty are hard to change in a short period time. If more sensitive evidence of a high-risk group and high-risk signals of suicide is provided, collaborative suicide prevention among agencies of public safety and public health agencies is possible.

For the empirical analysis, a nationally representative longitudinal sample of South Korean adults including information of late payments and suicidal behavior was used. Specific research questions are whether late payments are positively related to suicidal behavior, not explained by previous psychological health, and whether late payments are related to a greater risk of suicidal behavior independently from other socioeconomic characteristics.

## 2. Materials and Methods 

### 2.1. Study Population

Data were derived from the Korean Welfare Panel Study (hereafter, KOWEPS), which is an ongoing longitudinal study of a nationally representative sample of community-dwelling Korean adults, administered by the Korean Institute of Social and Health Affairs and the Social Welfare Research Institute of Seoul National University. In 2006, face-to-face interviews were first conducted for 24,463 participants who were at the age of 15 or older but not in middle or high school, from 7072 households sampled using a two-stage stratified cluster design. Annual follow-up surveys have been performed. In 2012, 1610 households were added in the panel to compensate for loss to follow up. So far, the first 10 waves of the KOWEPS were publicly released. This study is a secondary analysis of publicly available de-identified data, which are exempt from Institutional Review Board approval. The datasets and the user guide are publicly available at the website (http://www.koweps.re.kr) [20]. 

The present study used the 6th (2011) to 10th (2015) waves of data since the questionnaire of suicidal ideation and attempts was first included in 2011. We excluded Medicaid beneficiaries, who are not required to pay National Health Insurance (NHI) premium and pooled 10,596 participants who were included in the 2011 sample and 3350 participants who were newly recruited in 2012. Among the 11,725 participants who completed the next three consecutive waves of survey (9093 participants from the 2011 sample and 2632 participants from the new sample), observations with missing values were excluded, which left 10,988 individuals in the final analytic sample.

### 2.2. Suicidal Ideation and Attempts in the Past Year

As outcome measures, suicidal ideation and attempts during the past year were assessed by a questionnaire as follows: ‘Have you seriously considered suicide (ideation) at any time in the past year?’ and ‘Have you tried to commit suicide (attempt) at any time in the past year?’ Participants were required to provide a yes/no response for each question. A response in 2014 for the existing panel members and a response in 2015 for the newly recruited members were used for this study.

### 2.3. Late Payments

Paying late was our key explanatory measure. This was assessed with late payments of utility bills and NHI. The survey asks participants as follows: ‘Have you ever paid utility bills late in the last calendar year because of economic difficulty?’ and ‘Have you ever paid NHI premiums late in the last calendar year because of economic difficulty? We assessed whether a respondent had a late payment on either utility bills or NHI premium in each year. Then, the frequency of late payments was counted at the three consecutive waves (from 2012 to 2014 for the existing panel members and from 2013 to 2015 for the newly recruited members) and was categorized into three groups (never, once, and twice or more).

### 2.4. Covariates 

The past suicidal ideation and attempts at baseline (2011 or 2012) were measured as confounding factors because suicidal thought and intention might have caused late payments. The survey asked the existing panel members in 2011 and the newly recruited participants in 2012 about lifetime suicidal ideation and attempts, which precedes the measurement of late payments. Depression at baseline was adjusted for with the Center for Epidemiological Studies Depression Scale 11 (CES-D): ‘My appetite was poor’, ‘I felt I was just as good as other people’, ‘I felt depressed’, ‘I felt that everything I did was an effort’, ‘My sleep was restless’, ‘I felt lonely’, ‘I enjoyed life’, ‘People were unfriendly’, ‘I felt sad’, ‘I felt that people disliked me’, and ‘I could not get going’. Four-point scale responses (0 = one day; 1 = 3 days; 2 = 4–5 days; 3 = 4–6 days) for 11 questions were summed and a score of 9 was used as the cutoff for depression. 

Socio-demographic characteristics were adjusted for based on the 2013 survey (the 2014 survey for the newly recruited members) as follows: Gender (men, women), age (continuous variable), spousal status (with spouse, no spouse), occupational characteristics (regular employee snd self-employed, non-regular employee, not working), educational level (less than middle school, high school, better than college), and log transformed (equalized household) income. Current health status was assessed using self-reported health status (bad: Bad or very bad, not bad: Moderate, good, or very good) and number of chronic diseases (e.g., hypertension, diabetes, etc.) (none, one or more). 

### 2.5. Statistical Analysis

To assess the relationship between late payment and suicidal thoughts/behavior, multivariable logistic regressions were performed. First, each outcome measure of suicidal ideation and attempts was regressed adjusting for all the aforementioned covariates except for socioeconomic characteristics. Next, to investigate the independent prognostic importance of late payments, socioeconomic characteristics were further adjusted for. Longitudinal sampling weight was employed, and clustered standard error was estimated. For all analyses, Stata (version 12.0/SE) (StataCorp LP., College Station, TX, USA) was used.

## 3. Results

### 3.1. Descriptive Characteristics

Table 1 presents characteristics of the study population. Among the total of 10,988 respondents, 92.9% had no late payments, 5.2% one late payment, and 1.8% two or more late payments. Having two or more late payments was more prevalent in socio-economically disadvantaged groups such as the group of non-regular workers and the lowest-income group (4.8%). Those with bad self-reported health (2.8%) compared to good self-reported health (1.8%) and those depressed at the baseline (4.9%) compared to no depression (1.7%) had higher prevalence of having two or more late payments. Participants who reported lifetime suicidal thoughts and behavior at baseline had higher prevalence of having two or more late payments than those without lifetime suicidality (e.g., 7.4% vs. 1.9% for past suicide attempts).

### 3.2. Prevalence of Suicidal Ideation and Attempts by Individual Characteristics

Table 2 shows the prevalence of suicidal thoughts and behavior. The proportion of people with suicidal ideation was 2.66% in the sample and was higher in more disadvantaged groups. Individuals with two or more late payments (8.1%) had more than three times higher risk of suicidal ideation than those without late payments (2.4%). Among those who were not working, 3.48% reported they had thought about suicide in the past year whereas 1.91% of regular workers/self-employed did. Among people in the lowest income group, 7.19% reported they had thought about suicide, which was higher than in the highest group. 

In the sample, 0.11% of respondents reported they attempted a suicide during the preceding year. Those paying late twice or more showed an approximately 18 times higher proportion of suicide attempts in comparison with those without late payments (0.92% vs. 0.05%). Among high school graduates, 0.17% reported they had attempted suicide whereas the proportion in college graduates was only 0.01%. In the lowest-income group, the proportion of people that reported suicide attempts was 0.39%, which was higher than in the highest group. 

### 3.3. Relationship Between Suicidal Ideation and Attempts and Individual Characteristics

Models 1 and 2 of Table 3 present the estimates of the associations between suicidal ideation and late payments. Model 1 adjusted for all covariates except for economic status variables. This model showed that late payments were positively related to risk of suicidal ideation. Adults with two or more late payments had 2.32 times (95% CI: 1.39–3.86, *p* = 0.00184) increased the odds of suicidal ideation compared to having no late payments. Model 2 further adjusted for socio-economic status, which attenuated the relationship between late payments and suicidal ideation. The odds ratio of suicidal ideation for having one late payment was 1.50 (95% CI: 0.96–2.34, *p* = 0.0743), and having two or more late payments was related to a 2.11-times increased odds of suicidal ideation (95% CI: 1.22–3.65, *p* = 0.00858).

Estimates of the associations between risk of suicide attempts and late payments are presented in Models 3 and 4 in Table 3. Model 3 showed that risk of suicide attempts was positively associated with paying late. In comparison with having no late payments, having late payments was related to higher likelihood of suicide attempts (OR: 6.77, 95% CI: 2.03–22.58, *p* = 0.00262 for paying late once; OR: 10.99, 95% CI: 2.50–48.26, *p* = 0.00218 for paying late twice or more). Model 4 further adjusted for socio-economic status, which attenuated the relationship between late payment and suicidal attempts. The odds ratio of suicide attempts decreased to 5.46 (95% CI: 1.82–16.39, *p* = 0.00329) for people with one late payment and 7.44 (95% CI: 2.89–19.20, *p* = 0.00011) for those with two or more late payments.

## 4. Discussion

The findings show the following: First, the risk of suicidal ideation and attempts increased when they experience late payments, regardless of previous psychological health status. Second, adjusting other socio-economic characteristics did not change the relationship between late payments and suicide ideation/attempts, which means the independent effect of late payments on suicide behavior. With these findings, it can be suggested that late payment is an important factor, preemptively signaling suicide behavior with serious consequences in health and life.

This study provides an implication in crisis and emergency management from a mental health point of view. Like early detection and intervention is emphasized for effective management of public health crises, such as infectious disease outbreak, more efforts to monitor and respond to early signals of psychiatric problems need to be made in order to effectively reduce the mental health risks such as suicide-related behaviors [21]. In this sense, the evidence that late payments independently increase the risk of suicidal behavior is important because such small but accumulating financial difficulty can become an important stressor threatening mental wellbeing. 

Our findings are consistent with prior research on indebtedness. Studies in Finland, the US, and the UK found debt problems were related to increased risk of suicidal ideation [22,23,24]. However, few addressed the relationship between indebtedness and suicidal attempts in a general population. An exception is provided in the study in Finland, which found that indebtedness was not related to increased risk of suicide attempts using cross-sectional data [23]. The discrepancy in the findings between the previous work and the present study is probably related to differences in measurement of indebtedness. For example, whereas the previous study measured indebtedness based on cross-sectional data, the present study used the frequency of indebtedness. That is, repeated negative events may better reflect the financial difficulties that someone is experiencing [11]. In addition, by using longitudinal data and adjusting for history of psychological problem, the present study lessened the impact of possible confounding as compared to previous work.

A prior study on financial hardship showed similar results. For instance, economic hardship that was measured as the distance between the level of habit and actual consumption significantly predicted the trend of suicide rate [6]; when the consumption was lower than as it used to be, the disadvantageously changed condition affected an individual’s psychological wellbeing. It is plausible that financial difficulty prohibits one from purchasing goods and services, which is critical for satisfying the needs in daily living, and it may be a cause of increased unhappiness and suicidal thoughts [25,26]. In a similar vein, individuals having trouble paying bills may be afraid of or bothered by using basic necessities such as water, the lights, and heating, and it would increase distress and anxiety [19,22,27,28,29]. Further, such negative effects would even let them feel that there is no way out [10] if late payments continue. 

Considering a clear need to distinguish attempters from those who ideate, it needs to be noted that late payments appeared to be more strongly related to suicide attempts compared to suicide ideation in the present study. This especially implies a certain group of people is vulnerable to making a suicide attempt. Similar to our finding, prior work found that being unemployed was strongly tied to suicide attempts as opposed to ideation, whereas levels of depression and anxiety were similar in ideators and attempters [30,31]. A question may be raised why socioeconomic factors such as unemployment and late payments are more tied to suicide attempts. A potential explanation is that social stigma and prejudiced view regarding problem debt might prevent people with financial problems from seeking help or support [17,32], which inflates their risk of suicide attempts. 

Our study contributes to extend the previous research by focusing on late payment as an indicator of aggravating financial difficulty, and by considering both suicide thoughts and attempts, which can provide detailed empirical information of high-risk group for suicide prevention. For instance, community agencies in welfare and mental health in South Korea can take a joint vigilance action for the residents when their repeated late payment is reported by local financing agency.

From the policy perspective, our study also provides evidence that information on late payments could be used to act on high suicide burden. For example, several local governments in South Korea began to use information on late payments for finding out and helping disadvantaged households in 2016. We expect that the findings of the present study would help develop more effective interventions to prevent suicide that is related to economic problems. Furthermore, protection policies may be crucial to reduce the unintended harmful impact of late payments on suicidal behavior [33]. In addition, further policies to alleviate the negative influence of problems that may be associated with indebtedness, such as changes in employment status, may need to be considered [34], but the limited number of studies on determinants of household indebtedness restricts interpretation of our findings.

There are limitations to this analysis. First, the data of suicidal ideation and attempts included in the analysis were collected from self-reported survey. People who paid late bills and ideated/attempted suicide were less likely to participate in the survey, which may lead to an underestimation of the relationship between late payments and suicidal behavior. In addition, longitudinal studies have a common issue of selection bias due to differential loss to follow-up. In particular, people who delayed payments and ideated or attempted suicide were more likely to leave the follow-up. This could have caused a less strong positive association between late payments and suicidal behavior. Next, although a variety of confounding factors including depression and medical illness, which contribute to suicidal behavior as well as economic difficulties, were adjusted for by using longitudinal data, the observed relationship between late payments and suicidal behavior may not be causal. A third factor that was not considered may have been related to both the development of risk of suicidal behavior and late payment. Thus, cautious interpretations of the results are recommended. In addition, the confidence intervals of late payments were wide, which may be related to low occurrence of suicidal behavior and small sample size. Interpretations of the results should be cautious. Further research based on a large sample is needed to confirm and expand on these findings. Lastly, suicide attempts are not necessarily aimed at death. There may be the differences in intentions of suicide attempts. More disadvantaged people may have died by suicide without any record of suicide attempts, which may result in an underestimation of the association between late payments and suicidal behavior.

## 5. Conclusions

The findings of the negative association between suicide and economic conditions such as unemployment and low income have been accumulated. The remarkably high suicides in South Korea call for more preemptive efforts to look for a risk factor not only with a strong association with suicidal behavior, but also a potential for early detection and intervention of suicide behavior. As a response, this study investigated the impact of deteriorating economic condition in an individual’s daily life with a special focus on late payment of monthly bills by using longitudinal data. This study found the strong relationship between late payments of monthly bills (i.e., utility and health insurance) and suicidal ideation/attempts among the Korean population. The findings strongly supported the importance of late payments in predicting the risk of suicide behavior. Collaborative efforts in economic and health sectors are needed to develop preemptive support and involvement strategies to reduce suicide behavior. 

## Figures and Tables

**Table 1:** General characteristics of study population.

Late Payment		Overall		Non		One		Two+		*p*-Value
		N	%	N	%	N	%	N	%	
Total (*N* = 10,988)		10,988	100	10,212	92.9	576	5.2	200	1.8	
Sex	Male	4705	42.8	4368	93.3	243	4.6	94	2.0	0.66
	Female	6283	57.2	5844	93.2	333	5.0	106	1.9	
Age	20–39	2217	20.2	2061	94.0	114	4.3	42	1.7	0.05
	40–64	4555	41.5	4138	92.3	295	5.3	122	2.4	
	65 or older	4216	38.4	4013	94.9	167	4.0	36	1.1	
Marital status	Married	7551	68.7	7117	94.6	333	3.9	101	1.4	<0.001
	Single	3437	31.3	3095	90.1	243	6.8	99	3.1	
Regular employee/self-employed		4011	36.5	3803	95.0	158	3.6	50	1.4	<0.001
	Non-regular	2729	24.8	2440	89.1	208	7.7	81	3.2	
	Not working	4248	38.7	3969	94.1	210	4.2	69	1.7	
Education	<Middle school	4708	42.9	4423	92.9	219	5.0	66	2.0	<0.001
	High school graduates	3108	28.3	2791	90.6	228	6.5	89	2.9	
	College graduates	3172	28.9	2998	95.3	129	3.5	45	1.2	
Income	Q1	2747	25.0	2520	87.9	161	7.3	66	4.8	<0.001
	Q2	2747	25.0	2433	86.2	221	9.2	93	4.6	
	Q3	2750	25.0	2575	93.8	150	5.2	25	1.0	
	Q4	2744	25.0	2684	98.3	44	1.3	16	0.4	
Self-reported health	Good	8677	79.0	8070	93.5	451	4.6	156	1.8	0.005
	Bad	2311	21.0	2142	91.3	125	5.9	44	2.8	
Chronic disease	0	4766	43.4	4364	92.7	293	5.2	109	2.1	0.12
	≥1	6222	56.6	5848	94.0	283	4.3	91	1.7	
Depression at baseline	No	9646	87.8	9026	93.8	468	4.5	152	1.7	<0.001
	Yes	1342	12.2	1186	87.5	108	7.7	48	4.9	
Past ideation	No	9827	89.4	9207	94.0	467	4.4	153	1.6	<0.001
	Yes	1161	10.6	1005	86.7	109	8.5	47	4.8	
Past attempts	No	10,859	98.8	10,108	93.4	558	4.7	193	1.9	<0.001
	Yes	129	1.2	104	78.3	18	14.4	7	7.4	

Late payments were measured from 2012 to 2014 (from 2013 to 2015 for the newly recruited members).; *p*-values of the Chi-square test compare the prevalence of suicidal behavior across the different groups; age and income were used as continuous covariates in multivariable analyses; depressed condition, past ideation, and past attempts were measured based on the 2011 survey (the 2012 survey for the newly recruited members), and dependent and other independent variables were measured based on the 2013 survey (the 2014 survey).

**Table 2:** Proportion of individuals with suicidal ideation and attempts.

Variables		Ideation		Attempts	
		%	*p*-Value	%	*p*-Value
Total (*N* = 10,988)		2.66		0.11	
No. of late payment	0	2.39	<0.0001	0.05	<0.0001
	1	5.76		0.59	
	>1	8.10		0.92	
Sex	Male	2.29	0.021	0.04	0.0779
	Female	2.99		0.14	
Age	20–39	1.57	<0.0001	0.04	0.4761
	40–64	2.75		0.11	
	65 or older	4.45		0.11	
Marital status	Married	2.14	0.0017	0.06	0.0596
	Single	3.88		0.17	
Employment	Regular employee/self-employed	1.91	0.0052	0.04	0.2825
	Non-regular	2.94		0.09	
	Not working	3.48		0.16	
Education	<middle school	4.80	<0.0001	0.13	0.0604
	High school graduates	3.00		0.17	
	College graduates	1.35		0.01	
Income	Q1	7.19	<0.0001	0.39	0.003
	Q2	3.65		0.07	
	Q3	1.91		0.10	
	Q4	1.30		0.00	
Self-reported health	good	2.01	<0.0001	0.05	0.0001
	bad	7.40		0.37	
Chronic disease	0	1.67	<0.0001	0.02	0.0003
	≥1	3.94		0.19	
Depression at baseline	No	1.98	<0.0001	0.02	<0.0001
	Yes	9.71		0.78	
Past ideation or attempts	No	1.95	<0.0001	0.06	<0.0001
	Yes	8.97		2.67	

Late payments were measured from 2012 to 2014 (from 2013 to 2015 for the newly recruited members).; *p*-values of the Chi-square test compare the prevalence of suicidal behavior across the different groups; age and income were used as continuous covariates in multivariable analyses; depressed condition, past ideation, and past attempts were measured based on the 2011 survey (the 2012 survey for the newly recruited members), and dependent and other independent variables were measured based on the 2013 survey (the 2014 survey).

**Table 3:** Multivariable logistic regression results.

Variables	Model 1	Model 2	Model 3	Model 4
	Ideation	Ideation	Attempts	Attempts
	OR (95%CI)	OR (95%CI)	OR (95%CI)	OR (95%CI)
Number of late payment = 1 vs. never	1.64 *	1.50	6.77 **	5.46 **
	(1.05–2.55)	(0.96–2.34)	(2.03–22.58)	(1.82–16.39)
Numebr of late payment = 2 ^+^ vs. never	2.32 **	2.11 **	10.99 **	7.44 ***
	(1.39–3.86)	(1.22–3.65)	(2.50–48.26)	(2.89–19.20)
Women vs. men	1.01	0.98	2.52	2.43
	(0.80–1.28)	(0.74–1.30)	(0.42–15.09)	(0.33–17.94)
Age	1.05	1.02	0.99	0.96
	(0.97–1.12)	(0.95–1.09)	(0.68–1.44)	(0.68–1.36)
Age square	1.00	1.00	1.00	1.00
	(1.00–1.00)	(1.00–1.00)	(1.00–1.00)	(1.00–1.00)
No spouse vs. with spouse	1.65 **	1.60 **	1.08	1.25
	(1.22–2.22)	(1.19–2.13)	(0.20–5.90)	(0.30–5.21)
Non-regular vs. regular/self-employed		1.01		0.77
		(0.66–1.55)		(0.17–3.61)
No working vs. regular/self-employed		0.95		1.08
		(0.62–1.46)		(0.13–9.10)
Less than middle school vs. college		1.70 *		1.59
		(1.01–2.89)		(0.10–25.16)
High school vs. college graduates		1.75 **		7.77
		(1.25–2.44)		(0.88–68.59)
Log(income)		0.70		0.42
		(0.46–1.05)		(0.17–1.03)
Self-reported health: bad vs. others	2.05 ***	2.03 ***	2.23	2.67
	(1.55–2.71)	(1.53–2.68)	(0.63–7.85)	(0.80–8.92)
Chronic disease: 1 ^+^ vs. none	1.42	1.40	8.84 *	8.97 *
	(0.99–2.03)	(0.99–1.97)	(1.34–58.58)	(1.34–59.93)
Depressed at baseline vs. no	2.54 ***	2.48 ***	13.41 ***	13.96 **
	(1.78–3.62)	(1.65–3.73)	(3.19–56.38)	(2.77–70.27)
Past ideation/attempts at baseline vs. no	3.13 ***	3.07 ***	7.94 *	8.41 **
	(2.14–4.59)	(2.08–4.53)	(1.39–45.25)	(2.25–31.49)
Observations	10,988	10,988	10,988	10,988

OR: Odds Ratio; 95% CI: 95% confidence interval; *** *p* < 0.001, ** *p* < 0.01, * *p* < 0.05, ^+^ *p* < 0.1; Late payments were measured from 2012 to 2014 (from 2013 to 2015 for the newly recruited members), depression, past ideation/attempts from the 2011 (2012) survey, and dependent and other independent variables from the 2013 (2014) survey.
